# Military Anti-Tuberculosis Program Perfected

**Published:** 1918-12

**Authors:** Philip P. Jacobs

**Affiliations:** Assistant Secretary


### Military Anti-Tuberculosis Program Perfected.

BY PHILIP P. JACOBS, ASSISTANT SECRETARY.

Plans for a complete program for the Prevention of Tuberculosis in the army have been perfected by the National Association for the Study and Prevention of Tubercolosis working in cooperation with the Surgeon General, the Y. M. C. A., and other agencies. The program will include not only a follow-up for every man discharged on account of tuberculosis, but a thorough-going health educational campaign among the soldiers.

Prior to the first draft the National Association began to outline a preventive campaign. Owing to the magnitude of the task and the many practical delays in perfecting and applying the details of this scheme, the results were not as encouraging as might be expected. This was due to the fact that the report of names of men rejected by the draft on account of tubercolosis was inadequate, the slowness of the machinery in getting under way, and the many difficulties in determining the status of the men.

Inasmuch as enlisted or drafted men do not become accepted soldiers until after their probationary period lasting from three to six months in the various services, the Government assumes no responsibility for the after-care of those whose health breaks down during that period. Hence, this problem belongs to the civilian boards of health and the unofficial health organizations.

The National Asociation program falls into two main divisions: (a) Follow up work, and (b) educational work. The first obstacle to the follow-up program was section eleven of the Selective Service Regulations regarding the second draft which forbids giving a record of a man's condition to anyone except certain designated officials. The National Asociation officers, however, placed before the War Department the importance of this work and were influential in persuading them to open the records of rejected men to state and local Boards of Health throughout the country, through the United States Public Health Service and the Council of National Defense.

Inasmuch as the above section of the regulations does not apply to men dismissed from training camps after they have passed draftboards, the Asociation arranged with the Surgeon General and the division surgeons in camps to receive the names of all men thus dismissed. These lists are divided up by states and forwarded to state associations and state Boards of Health for follow-up work. Where men are referred to localities where there are not at present facilities for this follow-up work, the Association will use its good offices to promote the establishing of such facilities.

In the meantime, the Medical Department of the Army has perfected its machinery for weeding out these tuberculosis cases. Every man passed by the draft board after going into camp is examined by the Regimental Surgeon, re-examined by a tuberculosis board and then if suspected of tuberculosis, again examined by a tuberculosis expert. This follows a general policy mapped out and recommended by the National Association.

A large number of men have already been accepted into the service who were known to be tuberculous, many of them formerly inmates of tuberculosis sanatoria. Part of the Association's work has been to get in touch with every tuberculosis sanatorium and dispensary in the country and compile lists of all recent male inmates of draft age, giving the history of their cases and whether or not it was known if they were in the army at present. Hundreds of such names have already been received. This data is forwarded to the training camps, the men are located and the results are reported back to the sources of information.

Furthermore, the Association has sent a letter to all of its fifteen hundred local cooperating agencies giving the provisions of the second draft and urging that these agencies procure the names and addresses of all men of military age in their section who are known to have tubercuosis; get in touch with these men and arm them with the necessary affidavits to prevent, if possible, their being passed by the draft board, and recommend to the local draft boards the names of the approved tuberculosis experts in their section.

The Association is also cooperating with the Surgeon General's office to aid the Government in providing sanatoria for those men who have been discharged from the service on account of tuberculosis after their probationary period has expired. All full-fledged soldiers and sailors returned from France or other stations will be cared for as near to their own homes as possible in sanatoria accomodations provided by the Government. The

Government intends to utilize as far as possible existing institutions.

From the United States Marine Corps the National Association has secured each month a report of men rejected for tuberculosis from all its recruiting stations, and these men will receive the regular follow-up attention.

From the second or educational division of the program it is hoped to derive the greater ultimate good by the establishment of fundamental preventive measures among the well.

The National Association is interested in any kind of an educational campaign among the men in the various military camps that will tend to promote interest and information with regard to the control and prevention of cummunicable diseases, and toward the promotion of public and individual health in general. In the mobilization of such large numbers of men in various camps throughout the United States there have developed an unusual number of somewhat serious epidemics of colds, coughs, pneumonia, measles and various other respiratory and communicable diseases. That all of the diseases can be controlled by education and by the exercise of adequate public health measures has been clearly demonstrated in the civilian population throughout the United States. Most of these epidemics are spread through ignorance and carelessness. It is inevitable where large numbers of men from all walks of life and with all possible diseases and variations of physical habits are thrown together in somewhat uncomfortable and crowded living conditions, that there will be an immediate increase in the amount of sickness from communicable diseases. It must be obvious, however, to even the most superficial observers, that if these men can be taught to maintain a reasonable standard of personal hygiene and can be given a knowledge of the methods and principles of the control of communicable diseases a rapid diminution in the sickness rate will follow.

In cooperation with the Educational Committee of the National War Work Council of the Y. M. C. A., the National Association will furnish a number of stock lectures dealing with tuberculosis together with lantern slides to illustrate them. It will also arrange to put the educational secretaries of each of the camps in touch with the public lecturers in and around their respective camps. The Asociation has requested the War Department to give careful consideration to the desirability of appointing one or more special officers detailed to lecture on tuberculosis and allied health subjects in all of the army camps throughout the country.

The Association has prepared a special circular entitled "Red Blood," giving in brief and attractive form a message to the soldier relative to personal fitness, a heath "Don't Card;" and a Public Health Manual may also be distributed, the latter being a text book of personal hygiene.

The Association will also arrange to distribute through the departmental executives of the Y. M. C. A. a number of special tuberculosis exhibits known popularly as "The Parcel Post lantern slides will be used.

The National Association Field Secretary, Dr. Pattison, is visiting the training camps and supervising this educational work.

